# The mean and peak physical demands during transitional play and high pressure activities in elite football

**DOI:** 10.5114/biolsport.2023.112968

**Published:** 2022-02-04

**Authors:** Lukasz Bortnik, Joost Burger, David Rhodes

**Affiliations:** 1 Football Performance Hub, Institute of Coaching and Performance, University of Central Lancashire, United Kingdom; 2 Catapult Sports, Melbourne, Australia

**Keywords:** Football, Transitions, Counterattacks, High pressure, Peak demands, Worst-case-scenario

## Abstract

The aim of the present study was to establish the effect of transitional activities (TA) on physical metrics. Global Positioning System technology was utilized on 23 elite outfield footballers over 10 games to quantify absolute metrics per minute such as total distance (TD; m · min^-1^), sprint distance (SD; m · min^-1^), the number of high-intensity accelerations and decelerations (A+D; n · min^-1^), and high-speed running distance (HSRD; m · min^-1^). TD – total distance; HSRD – high-speed running distance; SD – sprint distance and high-intensity acceleration distance (Acc B3 Dist) were also quantified. Metrics were observed in relation to 4 TA’s commonly observed in football matches. Positive Transitions (PT), Negative Transitions (NT), Fast Attacks (FA) and High Pressure Activities (HP). Main effects for transition and game were observed. Comparisons were also made between 90 minute averages and transitional mean scores. NT displayed the highest TD (m · min^-1^) when compared to other TA’s (p ≤ 0.05). Observation of SD (m · min^-1^) for all transitions highlighted higher outputs when in PT (p ≤ 0.05). HP TA displayed the lowest output in all metrics (p ≤ 0.05), except high-intensity accelerations and decelerations A+D (n · min^-1^). The mean average and peak average outputs for TA and 90min average detailed elevated physical outputs across all metrics. Absolute physical metrics are increased when observing transitional play, representing the maximum physical exposure that athletes experience in games. This knowledge should be utilized when implementing high-velocity exposures within a weekly microcycle, to best prepare players for match play.

## INTRODUCTION

Soccer match play consists of short bouts of high intensity linear and multidirectional activities, interspersed with longer recovery breaks at lower intensity [1]. It has been shown that both physical and technical demands within the game of soccer have evolved substantially over the past decade [1–2]. Training design should integrate physical, technical and tactical aspects collectively [3] and reflect increased overall demands in order to successfully prepare players for high-intensity periods, which are crucial for the game outcome [4–5]. Modern wearable technology, including global positioning systems, offers a valid, reliable, practical and time-efficient solution for practitioners to quantify players’ external load and measure athletes’ movements in team sports [6]. Quantifying load during match play, provides a valuable reference for training load prescription [4]. However, literature currently focuses around whole game data with limited evidence analysing transitional play that often exposes players to the highest external loads within the modern game [7].

Observing game play identifies changes in running performance and match intensity during the game. Training design based on whole game data underestimates the physical and technical-tactical demands of high-intensity periods experienced by players [8–9]. These periods will also exhibit changing demands on specific positions within the team. Thus, posing the question as to whether prescribing training based on whole game data best prepares our athletes for the highest demands experienced during game play. Despite different terms being utilised in literature, the most intense periods during match play are called ‘worst case scenarios’ (WCS) [5, 7, 10]. Understanding the maximal physical demands during specific and short-lasting periods of match play, might allow coaches to prepare players more precisely for these high intensities [11].

WCS studies used durations from 10 seconds to 10 minutes and interestingly, shorter duration-specific periods have not been widely investigated [10]. The shorter the duration of the WCS the higher the intensity achieved in high-speed running, sprinting and high-intensity accelerations and decelerations [12–13]. Different methods for quantification of WCS exist and the rolling average method has been considered more accurate than fixed length method for measuring peak intensity during the periods of 1-, 3-, 5-, 10-min in professional soccer [12, 14, 15]. Another alternative and important approach to quantify WCS is to analyze actions when the ball is in play (BiP) [5] and/or quantify periods of repeated high-intensity efforts such as transitional activities [16]. In all available methods to measure WCS, the idea is the same – to identify the most intense periods of match-play in order to re-create the same physical stimulus in training and reach positive physiological outcome [17]. WCS or peak intensity demands is a multivariate concept, which must be properly addressed in practical settings. It rather represents an unstable benchmark today that is very difficult to be applied in practice and hence, it has been questioned [7].

It has been shown that players don’t achieve peak physical demands for different metrics simultaneously, which occur in different phases of the game [7]. There is lack of knowledge how the WCS information could be used for team physical conditioning purposes (small-sided games, technical-tactical drills, positional and running-based drills) such as volume/sets/duration and it is a challenge for practitioners to fully replicate and/or overload these match-play intense periods in training [13, 18]. There still remains a lack of knowledge of a number of the most intense passages in match play [15] as well technical-tactical actions that inevitably occur during these passages and which should not be omitted in the WCS analysis and training [18, 19]. Riboli and colleagues [20] described the distribution of the time spent at varied percentages of 1-min_peak_ for different physical metrics and identified that the majority of match activities overloaded average match demands, especially high-velocity activities. Despite new insights on match peak demands distribution, further insight is needed with regards what happens during these peak intensity passages considering a technical-tactical perspective. This would enable coaches and practitioners to understand how to best integrate physical and tactical aspects in training design to prepare players for maximal physical outputs in modern soccer game. Unfortunately, the current body of scientific research has focused on how to prescribe training based on only one physical variable (i.e. total distance per minute), which could limit specificity and consequently not fully reflect high physical stress players are exposed to in short and specific high-intensity passages [12].

As previously suggested [21], it is proposed that analysing transitions in play and high pressing in more detail and further exploring a multivariate meaning of the complex WCS concept. Offensive (defense-to-attack) transitions and defensive (attack-to-defense) transitions are short-duration specific actions that have been identified among the key five moments of play in soccer [11, 22–23]. Elite football teams in top leagues characterize a high tempo and fast attacking actions that last below 20 seconds [24]. Offensive transitions are characterized by high-speed actions with the goal to outnumber opponents, whereas defensive transitions by very quick re-organization of defensive shape [22, 25]. Rapid transitions from defensive to offensive moments of the games are counter-attacks [13], which surprise opposition’s imbalanced and disorganized defense [24], and have been found to be the most effective style of play for scoring goals [26]. Transitions are crucial phases of the game, since most goals and risks take place during these moments [23] and for this reason, they should be explored in more detail and analysed in relation to high-intensity and high-velocity activities that occur during these phases. In addition, high pressing in the offensive third was found to generate seven times more goals and create more goal scoring opportunities [25]. High pressure has been believed to be linked to high levels of fitness [21].

Regardless of the significance of TA’s within soccer match-play and the fact that they could be easily replicated in training, there are no studies that investigate physical demands during these phases [11], compare them to the 90-min demands and treat them as peak intensity periods as well as inform practice about specific physical targets to improve teams performance during their offensive and defensive activities. In contrast to WCS concept, TA’s have been well defined and directly linked to technical-tactical activities that occur simultaneously with other physically demanding activities within a modern soccer match play [22, 25, 27]. In addition, TA’s include a high context within as it represent moments when ball is in play, describe phases when team is either in (offensive actions) or out of the ball possession (defensive actions), and relates to tactical aspects such as offensive activities (counter-attack and fast attack) and defensive actions (attack-to-defense transition and high pressure). Therefore, the current study aims to determine the physical demand (total distance, high-speed running distance, sprint distance and high-intensity accelerations/decelerations) and frequency of transitional activities in games, comparing them to 90-minute match demands.

## MATERIALS AND METHODS

### Participants

Twenty-three elite footballers from the Polish premier league were included in the study. Players were classified according to playing position, resuting in the following number per position: center backs (*n* = 4), full backs (*n* = 5), central defensive midfielders (*n* = 2), central attacking midfielders (*n* = 2), central midfielders (*n* = 2), wingers (*n* = 5), and attackers (*n* = 3). Each game only included data from those players who played at least 60 min, since substitutes can have higher outputs than starting players likely as a result of pacing strategies [9, 11]. All players were competing in the 1^st^ Polish Division (Ekstraklasa) in season 2020–21. Players were presented with information of the project protocol and provided informed consent for the use of match data, in accordance with the Helsinki Declaration. All data was anonymised prior to data analysis to ensure player confidentiality. Ethical approval was provided by the host university.

### Procedures & Experimental design

Data was collected between August and November 2020, during which a total of ten official matches including one UEFA CL qualifier and nine Polish domestic league (Ekstraklasa) games were analysed. Players’ movements were captured by MEMS (10 Hz; Vector S7, Catapult Sports, Melbourne, Australia), which were worn in each game between the scapulae and contained within the playing jersey inside a pocket. The players were familiar with the use of these devices, wearing them usually in training and games. Devices were turned on 15 mins before the start of the match to get a better connection to the satellites. Each data was screened for satellite coverage and horizontal dilution of precision (HDOP) using an inclusion criterion of > 6 satellites and ≤ 1.0 respectively, which are in accordance to previous guidelines for acceptable GPS coverage [28]. Players wore the same device during each game to reduce the interunit variations. The reliability and validity of such technology has been previously presented [6, 29].

Variables analysed were selected based on previous publications [5, 11, 13]. Absolute distances covered per minute (m · min^-1^) in the following categories: total distance (TD), high-speed running distance (HSRD, > 19.8 km·h^-1^), sprint distance (SD, > 25.2 km·h^-1^), as well as the number of high-intensity accelerations and decelerations (A+D, > 3 m · s^-2^; n · min^-1^) were observed. Additional observed variables were absolute distances covered in the following categories: total distance (TD), high-speed running distance (HSRD), sprint distance (SD) and acceleration distance (Acc B3 Dist, distance with variations in running speed > 3 m · s^-2^). Following each match, transitions were identified and manually generated in the Catapult Vision video analysis system (Catapult Sports Ltd, Melbourne, Australia) by the club’s analysis team. These transitions were categorized as followed: positive transition (PT), negative transition (NT), fast attack (FA), and high pressure (HP). Identification of these transitional actions by the analysis team was completed utilizing the observational methodology REOFUT theoretical framework [30]. All analysts involved in the present study had previously completed training on the use of the REOFUT instrument and as part of the clubs game analysis protocols implemented this as part of their daily work. The inter and intra observer reliability of these methods has been previously well described in literature, identifying a good to high intra-inter reliability [24–25, 27, 31].

Positive transition (PT) described a counter-attack [24], which had the following characteristics: a) the possession starts by winning the ball in play, b) the progression towards the goal attempts to utilize the degree of imbalance right from start to the end with high tempo [25], c) the circulation of the ball takes place more in depth than in width and the intention of the team is to exploit the space left by the opponent when they were attacking, d) the opposing team does not have the opportunity to minimize surprise, reorganise their system and be prepared defensively. Negative transition (NT) was a reversed counter-attack (transition from attack-to-defense), which described defensive actions of the team against an opposition counterattack defined above [24]. Fast attack (FA) a) started by wining the ball in play or restarting the game, b) the progression towards the goal had a high number of penetrative and short passes, c) the circulation of the ball took place in width and depth and the intention of the team is to disorder the opponent with a reduced number of passes and high tempo, d) the opposing team had the opportunity to minimize surprise, reorganize his system and be prepared defensively [24]. High pressure (HP) occured when one or several offensive (attacking) players press the opposing team close to their penalty area (attacking third) to re-gain the ball and start a counterattack within the first 3 seconds of the possession (the defender(s) are always located within 1.5 m from the first attackers) [27].

Data from the Catapult vision software was then downloaded and integrated into the manufacturer’s software package (*Openfield*, version 3.2.0) and finally exported into Microsoft Excel (Microsoft Corporation, USA) to calculate relative distances in selected categories for each transitional play. In addition to obtaining the mean average and peak average of each given metric per minute during TA’s. The transition mean average for selected metrics was calculated as the sum total of all TA’s, divided by their number. To obtain the transition peak average value for each metric, the highest values in 10 games were identified, and their average was calculated as the sum of all peak values during transitions, divided by their number.

### Statistical analysis

A descriptive analysis was performed and the results are presented as mean ± standard deviation (SD). Between-matches coefficient of variation (CV) values were calculated for transitions and the whole match (90-min) demands for total distance (m · min^-1^), high speed running distance (m · min^-1^), sprint distance (m · min^-1^), and number of accelerations/decelerations (n · min^-1^).

Statistical analyses were conducted using IBM Statistical Package for the Social Sciences (SPSS, Version 27.0, IBM Corporations, New York, USA) with the statistical significance accepted at the 0.05 level. A univariate analysis of variance (ANOVA) was conducted to quantify main effects for games and transitions. Interaction effects were also quantified, and any significant main effects associated with games and transitions were explored using post hoc pairwise comparisons. The assumptions associated with the statistical model were assessed to ensure model adequacy. To assess residual normality for each dependant variable, q-q plots were generated using stacked standardised residuals. Scatterplots of the stacked unstandardized and standardised residuals were also utilised to assess the error of variance associated with the residuals. Mauchly’s test of sphericity was also completed for all dependent variables, with a Greenhouse Geisser correction applied if the test was significant. Partial eta squared (η^2^) were calculated to estimate effect sizes for all significant main effects and interactions. As previously recommended [32], partial eta squared was classified as small (0.01–0.059), moderate (0.06–0.137), and large (> 0.138).

## RESULTS

Table 1 highlights the mean number of transitions that occur per game, accompanied with the standard error, min, max and confidence interval (CI). The frequency of each transition across the games analysed is displayed in table 2. Also, detailed is the percentage that each transition accounts for, the mean and standard deviation of each transition type and the peak duration of each transition across all games analysed.

**TABLE 1:** Mean ± SD, minimum and maximum count of transitions PT = Positive transition; NT = Negative transition; FA = Fast attack; HP = High pressure for each game at 95% confidence interval of difference (CI).

TRANSITIONS	
Mean ± SD	50 ± 11.1
Standard Error	3,52
Minimum	32
Maximum	68
CI	7.97

**TABLE 2:** Frequency of transitions PT = Positive transition; NT = Negative transition; FA = Fast attack; HP = High pressure depicted as a count and percent. and duration of transitions expressed as a mean ± SD and peak value across 10 official matches.

Transition	Frequency		Duration (s)	
	Count	Percent (%)	Mean ± SD	Peak
PT	137	28	10.9 ± 3.9^bcd^	24
NT	152	31	9.3 ± 3.4^a^	23
FA	130	26	10.0 ± 3.1^ad^	22
HP	77	16	9.8 ± 3.7^ac^	27

Note: Significant difference (p < 0.05) compared to the PT (a). NT (b). FA (c). and HP (d).

Displayed in table 3 is the mean, standard deviations and CI for total distance – TD (m · min^-1^), high speed running distance – HSRD (m · min^-1^), sprint distance – SD (m · min^-1^), and number of accelerations/decelerations – A+D (n · min^-1^) for mean and peak transitions and 90 minute averages for each of the listed metrics.

**TABLE 3:** Team mean ± SD and 95% confidence intervals for total distance – TD (m·min^-1^), high speed running distance – HSRD (m·min^-1^), sprint distance – SD (m·min^-1^), and number of accelerations/decelerations – A+D (n·min^-1^), across whole-match vs mean and peak transitions demands.

	TD		HSRD		SD		A+D	
	Mean ± SD	95%CI	Mean ± SD	95%CI	Mean ± SD	95%CI	Mean ± SD	95%CI
**90-min**	109.7 ± 3.7	107.1–112.3	7.7 ± 0.9	7.0–8.4	1.6 ± 0.5	1.2–2.0	0.76 ± 0.1	0.71–0.81
**Transitions (mean)**	203.0 ± 12.2	194.3–211.7	51.1 ± 9.1	44.6–57.6	15.1 ± 4.0	12.2–18.0	1.1 ± 0.2	1.0–1.2
**Transitions (peak)**	290.3 ± 30.8	241.2–339.4	164.7 ± 61.2	67.4–262.0	84.0 ± 41.8	17.5–150.5	4.7 ± 0.8	3.4–6.0

**FIG. 1:**
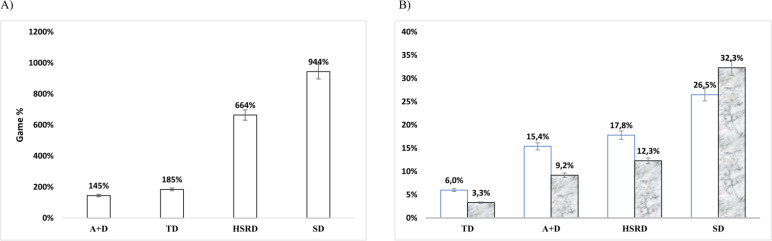
Transitions as percentage of the whole-match demands (90-min) (A) and the match-to-match variability for both transitions and 90 min (B) are shown for total distance – TD in m·min^-1^, high-speed running distance – HSRD in m·min^-1^, sprint distance - SD in m·min^-1^ and number of accelerations/decelerations A+D in n·min^-1^.

Figure 1 displays transitions as a percentage of the whole-match demands and details the match-to-match variability for transitions and 90 min game averages.

**FIG. 2:**
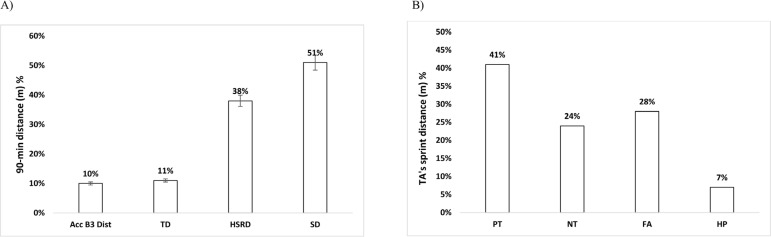
Total physical output during transitions PT = Positive transition; NT = Negative transition; FA = Fast attack; HP = High pressure as percentage of the whole-match output (90-min) (A) and sprint distance as percentage of the whole-transitions sprint distance (B) are shown for total distance (TD), high-speed running distance (HSRD), sprint distance (SD) and acceleration distance (Acc B3 dist).

Figure 2 represents total physical output during 4 transitions as percentage of the whole-match output and highlights sprint distance during transitions.

**FIG. 3:**
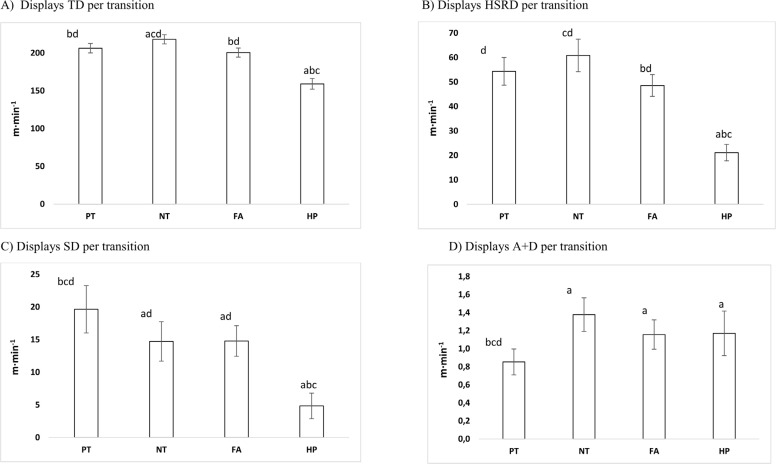
Comparisons between all transitions PT = Positive transition; NT = Negative transition; FA = Fast attack; HP = High pressure in a) mean total distance per minute (TD), b) mean high-speed running distance per minute (HSRD), c) mean sprint distance per minute (SD), and d) mean number of accelerations/decelerations per minute (A+D). **Significant difference (p < 0.05) compared to the PT (a), NT (b), FA (c), and HP (d).

Comparisons between all transitions in total distance – TD (m · min^-1^), high speed running distance – HSRD (m · min^-1^), sprint distance – SD (m · min^-1^), and number of accelerations/decelerations – A+D (n · min^-1^) can be seen in figure 3.

### Duration

There was a main effect of transitions on duration, *F*(3,456) = 9.997, *p* < 0.0005, partial η^2^ = 0.062. Negative transition (NT) duration displayed no difference in relation to fast attacks (FA) and high pressure (HP) (*p* = .142 and *p* = .198, respectively). Positive transitions (PT) duration was higher than all other transitions (*p* ≤ *0.05*).

### Total distance (TD; m · min^-1^)

There was a main effect of transitions, *F*(3,456) = 26.628, *p* < 0.005, partial η^2^ = 0.149 as well as games, *F*(9,456) = 2.010, *p* = 0.037, partial η^2^ = 0.038 for total distance. Negative transitions (NT) had higher m · min^-1^ than PT, FA and HP (*p* ≤ *0.05*). High pressure (HP) showed lower distance than all transitions (*p* ≤ *0.05*). See Figure 3 (Panel A).

### High-speed running distance (HSRD; m · min^-1^)

There was a main effect of transitions, *F*(3,456) = 12.244, *p* < 0.005, partial η^2^ = 0.075 for high speed running distance. Post hoc analysis showed that high pressure (HP) revealed difference from all other transitions (*p* ≤ *0.05)*. See Figure 3 (Panel B).

### Sprint distance (SD; m · min^-1^)

There was a main effect of transitions, *F*(3,456) = 5.488, *p* = 0.001, partial η^2^ = 0.035 for sprint distance. Multiple comparisons show that negative transitions (NT) were nearly equal to fast attacks (FA) (*p* = 0.974). Positive transitions (PT) showed difference from negative transitions (NT) and fast attacks (FA) (*p* = 0.014 and *p* = 0.019, respectively). Furthermore, PT were higher than high pressure (HP) (*p* ≤ *0.05*). High pressure (HP) was the lowest from all other transitions (*p* ≤ *0.05*). See Figure 3 (Panel C).

### Accelerations and decelerations number (A+D; n · min^-1^)

There was an interaction between games and transitions for high-intensity accelerations and decelerations, *F*(27,456) = 1.606, *p* = 0.029, partial η^2^ = 0.087. High pressures (HP) were nearly equal to fast attacks (FA) (*p* = .927). Positive transitions (PT) were lower than NT, FA and HP (*p* ≤ *0.05*). See Figure 3 (Panel D).

## DISCUSSION

The aim of the present study was to determine the physical demand (total distance, high-speed running distance, sprint distance and high-intensity accelerations/decelerations) and frequency of transitional activities in games, comparing them to 90-minute match demands. Main findings revealed that transitions occurred on average 50 times across ten official games, with a range of 32 to 68. Negative transitions (NT) were most frequent, followed by positive transitions (PT), fast attacks (FA), and high pressure (HP) activities. It is suggested that transitional strategy of the team and opponent would effect the number and type of transition seen in game play. Future research should consider tactical strategy of the team and opponent, which the present study failed to do. Positive transitions (PT) lasted significantly longer than other periods (around 11 secs), while mean duration of transitions was around 10 secs. Peak duration of all transitions lasted between 22–27 secs. Findings consistent with previous work detailing that most transitions performed were shorter than 20 seconds [24].

Negative transitions (NT) accumulated higher TD (m · min^-1^), while positive transitions (PT) generated highest SD (m · min^-1^). This has interesting implications on performance, particularly when considering previous literature detailing the importance of this metric in creating chances and scoring goals [25, 33]. Counter-attacks have been demonstrated to display high physical outputs often associated with high-velocity movements in to space with the objective of getting to the opponents box to create goal scoring opportunities [24, 33–36]. The context of these findings are important and practitioners need to consider the volume of exposure to high-velocity activities such as sprinting, accelerations, decelerations and high-speed running within training. That said, the present work doesn’t consider high-velocity distances covered and average time between transition in games in relation to these transitional activities. Analysis of this would enhance training design. Our findings showed that mean transition performance demonstrated around 16% match-to-match variability and transitions sprinting activities (SD; m · min^-1^) exhibited lower variability compared to the 90-min sprinting demands, findings consistent with literature [13, 37–38]. Thus, demonstrating the complexity of training design for footballers, due to the unpredictability of game play.

High pressure (HP) activities generated lower physical output in all metrics, except for A+D (n · min^-1^). It has been shown that defensive activities require players to actively engage in regaining the possession of the ball, squeeze space and block forward passes, which demands high-intensity actions such as high-intensity accelerations and decelerations [39]. Often these actions are initiated by the opposing teams movement, so become reactive high velocity actions (accelerations and decelerations) to put pressure on the ball quickly. Pressing high in the offensive zone (opposition half) is essential as nearly half of all winning ball turnovers, across various european leagues, are shown to create goal scoring opportunites [27, 35, 40–41]. Coaches could apply football-specific drills that utilize high pressure to induce a higher number of accelerations and decelerations in training without accumulating higher distances in different velocity bands. These rapid changes of speed are crucial in elite soccer and efficient and quick ability to accelerate, decelerate, change of direction have been linked to success in field-sport teams [2, 8] and soccer match result [42].

A novel concept in the present study was to directly compare players physical output during transitions directly to the 90-min demands. To the authors knowledge, this is the first study that investigates the mean and peak physical demands during TA’s. Considering a shorter than usually investigated time epochs for the WCS analysis, comparisons to other studies could be difficult. Findings detailed that transitions exceeded the 90-min physical demands in all variables, especially in high-velocity activities (SD; m · min^-1^) in both mean and peak transitions. High-velocity activities were 7–9-fold greater than the 90-min demands and nearly half of the game SD and HSRD occurred during TA’s. Presenting practitioners with considerations of how these demands are replicated in training to best prepare their athletes.

Interestingly, offensive actions such as positive transitions (PT) and fast attacks (FA) mostly contributed to sprint distance accumulated during transitional periods (41% and 28%, respectively) and high pressure (HP) activities accumulated the lowest sprint distance equal to only 7%. Contextually, how players are prepared during training would depend on the tactical focus of the teams approach to games. Over 90 minutes, players cover TD of around 119 m· min^-1^, HSRD of 7.5 m · min^-1^ and SD of 1.5 m · min^-1^ [43–44], which is consistent with our findings, especially in regards to high-velocity metrics. Physical output during 1-min peak passages (WCS) has been shown to be higher during which elite players cover TD (m·min^-1^) ranging from 167 to over 190, HSRD (m · min^-1^) from 38.3 to 60, and SD (m · min^-1^) around 10.6 [9, 12–14, 45]. In addition, a previous analysis of ball in play (BiP) short-lasting WCS window (30–60 s) showed that elite youth players cover a total distance of 200.9 (m · min^-1^), high-speed running distance of 68.3 (m · min^-1^) as well as perform 5 accelerations and 5.1 decelerations (n · min^-1^) [11]. These values are very similar to our findings across all mean metrics, but still lower compared to peak variables during this specific and short-lasting maximum outputs. Thus, contextualizing high-intensity metrics that occur in relation to short-duration blocks through a 90-min period is crucial for understanding training needs and best preparing athletes to minimize injury risk and increase performance [46–47].

Well-defined short-lasting passages (transitions) might offer new multivariate insights on high-intensity periods in modern football and allow practitioners to understand their impact on performance. High intensity periods (high velocity) with a clear tactical objective (i.e. transition from defense to offence) should be replicated in training to ensure specificity. It has been demonstrated that maximum speed activities over longer distances have not been adequately used in soccer [43]. In addition, tailoring training in a similar manner and utilizing principles of overload within a weekly training block has been shown to induce desired physiological adaptations in soccer players [3, 48–49].

Due to the peak demands displayed in the present study a key objective of training would be to reach maximum speed and generate higher sprint distances, practitioners could use offensive position-specific exercises as well as transitional games to do this. The main target during these games would be a very quick attack after the ball possession was recovered. The important condition, which should be applied, would be to create large spaces behind the defenders. Enabling the attacking team to exploit it and generate high-velocity movements with a tactical objective to finilize this action. Transitions could be effectively used to overload average match play intensity concurrently in different physical metrics. Practitioners could use isolated transitional drills (positive and negative) as well as conditioning transitional games (large-, medium-, and small-sided) in MD-4 and/or MD-3 sessions (midweek) to overload locomotor and mechanical demands and increase high-velocity stress on players [12, 43, 50]. It has been shown that large-sided games (LSG10) were the most appropriate mode of soccer-specific training (midweek) to reach similar intensity in many physical metrics and even over-stimulate (125%) sprint demands in relation to the 5- and 10-min peak match demands. In contrast, small-sided games (SSG5 and SSG6) were found to over-stimulate (150%) high-intensity accelerations/decelerations, but under-stimulate locomotive variables such as distance, high speed running, and sprinting [51]. Therefore, well balanced transitions to replicate the high-intensity / high-velocity demands of competition during team, individual and/or end-stage rehabilitation sessions could better prepare athletes for the game demands, increase performance and reduce the risk of injury. Consideration should be given to recovery taken after and before match play for such high-intensity activities, which have been linked to muscle fatigue and higher injury risk [49, 52–53]. Further work in this area is required. It is equally important to acknowledge that these demands would change in relation to positional requirements and individual player physical capability, willingness to run, coach style, opposition level, and other contextual factors [34, 38, 54–56].

The activity profile of elite soccer players within 90-min match play has been found to be highly dependent on the tactical role and playing position [19, 57]. Similar to the 90-min demands, positional differences in physical output has been found among different positions during varied WCS periods [12–13, 15, 44, 51]. The present body of work did not analyse positional differences in physical demands across different TA’s. Future work should consider these positional differences. Research analysing the impact of contextual variables (match location, match half, and match outcome) on WCS within a soccer match is scarce [13–15]. Contextual factors such as changes on the tactical and technical requirements of the match play most likely determine the physical output experienced by players during the most intense passages in modern football, especially for near maximum velocity activities [38]. It is important to note the present study was completed with one club over ten games (small sample), so future research should consider a larger sample across a number of teams across the league in a season, investigate physical differences between positions, identify impact of substitutes on transitions, compare training to match play transitional demands, and include additional contextual factors, such as formation, to enable individualization. This would allow conclusions to be drawn in relation to game demands in modern day football. The present study analysed only absolute metrics and it is noteworthy to emphasize the importance of monitoring players relative to their physical performance capacities. Absolute zones/metrics enable practitioners to compare players physical outputs, but do not show their individual physical characteristics [2, 58]. Particularly regarding high-velocity activities such as high-speed running and sprinting. Relative locomotor zones might be more appropriate to analyze players’ true workload during the most demanding passages of match play [59]. Hence, future work should also consider analyzing transitional play using individual velocity and accelerations thresholds.

## CONCLUSIONS

Understanding of the frequency, duration and type of transition that generates the highest physical absolute outputs is important to practitioners. Present literature describes and highlights the use of 90-min average data to inform how to best prepare athletes for modern game play within training. However, the present study demonstrates that these metrics are largerly increased when contextualized into transitional play, representing the maximum physical exposure that these athletes experience in games. Consideration of these maximal outputs informs practitioners of the physical demands footballers will face within given time frames during offensive and/or defensive activities. This knowledge should be utilized when implementing high-velocity/high-intensity exposures within a weekly microcycle to best prepare players for these high-intensity periods in match play. Caution should be taken as these findings are representative of one team within the Polish top league.

## Key points summary

– Transitions exceed 90-min physical demands in all metrics and expose players to maximum physical outputs.
– Coaches should develop training strategies that replicate these demands by placing conditions in training drills, manipulating pitch size and selecting appropriate number of players in order to best prepare athletes for competition, increase players performance and reduce injury risk.
– Since playing position/formation (e.g., 4-4-2, 4-3-3, 3-5-2, etc.)/playing style would most likely generate different physical metrics during TA’s, coaches and practitioners are encouraged to assess these differences to design and deliver optimal training programmes for all players according to their tactical roles and individual abilities.
– Offensive actions (counter-attacks and fast attacks) expose players to maximum velocity activities (sprint distance) and could be used in the midweek overload block for conditioning purposes.
– High pressure activities increase mechanical load on players and might be used to incrase accelerations and decelerations demands in team raining, top-up sessions and end-stage rehabilitation programmes.

## References

[1] Barnes C, Archer DT, Hogg B, Bush M, Bradley PS. The evolution of physical and technical performance parameters in the English Premier League. Int J Sports Med. 2014; 35(13):1095–1100. 25009969 10.1055/s-0034-1375695

[2] Harper DJ, Carling C, Kiely J. High-Intensity Acceleration and Deceleration Demands in Elite Team Sports Competitive Match Play: A Systematic Review and Meta-Analysis of Observational Studies. Sports Med. 2019; 49(12):1923–1947. 31506901 10.1007/s40279-019-01170-1 PMC6851047

[3] Stølen T, Chamari K, Castagna C, Wisløff U. Physiology of soccer: an update. Sports Med. 2005; 35(6):501–536. 15974635 10.2165/00007256-200535060-00004

[4] Anderson L, Orme P, Di Michele R. Quantification of Seasonal-Long Physical Load in Soccer Players With Different Starting Status From the English Premier League: Implications for Maintaining Squad Physical Fitness. Int J Sports Physiol Perform. 2016; 11(8):1038–1046. doi:10.1123/ijspp.2015-0672. 26915393

[5] Pollard BT, Turner AN, Eager R, et al. The ball in play demands of international rugby union. J Sci Med Sport. 2018; 21(10):1090–1094. 29559318 10.1016/j.jsams.2018.02.015

[6] Scott MT, Scott TJ, Kelly VG. The Validity and Reliability of Global Positioning Systems in Team Sport: A Brief Review. J Strength Cond Res. 2016; 30(5):1470–1490. 26439776 10.1519/JSC.0000000000001221

[7] Novak R, Impellizzeri FM, Trivedi A, Coutts AJ, McCall A. Analysis of the worst-case scenarios in an elite football team: Towards a better understanding and application. J Sports Sci. 2021; 39(16):1850–1859. 33840362 10.1080/02640414.2021.1902138

[8] Delaney JA, Scott TJ, Thornton HR, Bennett KJ, Gay D, Duthie GM, Dascombe BJ. Establishing Duration-Specific Running Intensities From Match-Play Analysis in Rugby League. Int J Sports Physiol Perform. 2015; 10(6):725–31. 26023738 10.1123/ijspp.2015-0092

[9] Lacome M, Piscione J, Hager JP, Carling C. Analysis of Running and Technical Performance in Substitute Players in International Male Rugby Union Competition. Int J Sports Physiol Perform. 2016; 11(6):783–792. 26658134 10.1123/ijspp.2015-0191

[10] Whitehead S, Till K, Weaving D, Jones B. The Use of Microtechnology to Quantify the Peak Match Demands of the Football Codes: A Systematic Review. Sports Med. 2018; 48(11):2549–2575. 30088218 10.1007/s40279-018-0965-6 PMC6182461

[11] Wass J, Mernagh D, Pollard B, Stewart P, Fox W, Parmar N, Jones B, Kilduff L, Turner A. A comparison of match demands using ball-in-play vs. whole match data in elite male youth soccer players. Science and Medicine in Football. 2020; 4(2):142–147.

[12] Martín-García A, Casamichana D, Díaz AG, Cos F, Gabbett TJ. Positional Differences in the Most Demanding Passages of Play in Football Competition. J Sports Sci Med. 2018; 17(4):563–570. 30479524 PMC6243617

[13] Riboli A, Semeria M, Coratella G, Esposito F. Effect of formation, ball in play and ball possession on peak demands in elite soccer. Biol Sport. 2020; 38(2):195–205. 34079164 10.5114/biolsport.2020.98450 PMC8139352

[14] Ferraday K, Hills SP, Russell M, Smith J, Cunningham DJ, Shearer D, Mcnarry M, Kilduff L. A comparison of rolling averages versus discrete time epochs for assessing the worst-case scenario locomotor demands of professional soccer match-play. J Sci Med Sport. 2020; 23(8):764–9. 31937507 10.1016/j.jsams.2020.01.002

[15] Oliva-Lozano J, Fortes VM., Muyor J. The first, second, and third most demanding passages of play in professional soccer: a longitudinal study. Biol Sport. 2021; 38(2):165–174. 34079161 10.5114/biolsport.2020.97674 PMC8139346

[16] Aranda R, Gonzalez-Rodenas J, Lopez-Bondia I, Aranda-Malaves R, Tudela-Desantes A, Anguera MT. “REOFUT” as an observational tool for tactical analysis on offensive performance in soccer: mixed method perspective. Front psychol. 2019; 10, 1476. 31316433 10.3389/fpsyg.2019.01476 PMC6610999

[17] McCall A, Pruna R, Van der Horst N, Dupont G, Buchheit M, Coutts A, Impellizzeri F, Fanchini M. Exercise-Based Strategies to Prevent Muscle Injury in Male Elite Footballers: An Expert-Led Delphi Survey of 21 Practitioners Belonging to 18 Teams from the Big-5 European Leagues. Sports Med. 2020; 50:1667–1681. 32676903 10.1007/s40279-020-01315-7 PMC7441050

[18] Carling C, McCall A, Harper D, Bradley PS. Comment on: “The Use of Microtechnology to Quantify the Peak Match Demands of the Football Codes: A Systematic Review”. Sports Med. 2019; 49(2):343–345. 30506338 10.1007/s40279-018-1032-z

[19] Bradley P, Evans M, Laws A, Ade J. ‘Context is King’ when Interpreting Match Physical Performances. Footb Med Sci. 2018; 24:42–45.

[20] Riboli A, Esposito F, Coratella G. The distribution of match activities relative to the maximal intensities in elite soccer players: implications for practice. Res Sports Med. 2021; (3):1–12. DOI: 10.1080/15438627.2021.1895788 33657944

[21] Wright C, Atkins S, Polman R, Jones B, Sargeson L. Factors associated with goals and goal scoring opportunities in professional soccer. Int J Perform Anal Sport. 2011; 11(3):438–449.

[22] Hewitt A, Greenham G, Norton K. Game style in soccer: what is it and can we quantify it? Int J Perform Anal Sport. 2016; 16(1):355–372.

[23] Lago-Peñas C, Gómez-Ruano M, Yang G. Styles of play in professional soccer: an approach of the Chinese Soccer Super League. Int J Perform Anal Sport. 2018; 17(6):1073–1084.

[24] Gonzalez-Rodenas J, Aranda-Malaves R, Tudela-Desantes A, Nieto F, Uso F, Aranda R. Playing tactics, contextual variables and offensive effectiveness in English Premier League soccer matches. A multilevel analysis. PLoS ONE 2020; 15(2): e0226978. 32069336 10.1371/journal.pone.0226978 PMC7028361

[25] Tenga A, Holme I, Ronglan LT, Bahr R. Effect of playing tactics on goal scoring in Norwegian professional soccer. J Sports Sci. 2010; 28:237–244. 20391095 10.1080/02640410903502774

[26] Lago-Ballesteros J, Lago-Peñas C, Rey E. The effect of playing tactics and situational variables on achieving score-box possessions in a professional soccer team. J Sports Sci. 2012; 30(14):1455–61. 22856388 10.1080/02640414.2012.712715

[27] Armatas V, Ampatis D, Yiannakos A. Comparison of the effectiveness between counter-attacks and organized offences in Champions League 2002–03. 1st International Scientific Congress in Soccer. Trikala: Greece. 2005. p. 8–10.

[28] Malone JJ, Lovell R, Varley MC, Coutts AJ. Unpacking the Black Box: Applications and Considerations for Using GPS Devices in Sport. Int J Sports Physiol Perform. 2017; 12(2):18–26. 27736244 10.1123/ijspp.2016-0236

[29] Johnston RJ, Watsford ML, Kelly SJ, Pine MJ, Spurrs RW. Validity and interunit reliability of 10 Hz and 15 Hz GPS units for assessing athlete movement demands. J Strength Cond. Res. 2014; 28:1649–1655. 24276300 10.1519/JSC.0000000000000323

[30] Collins K, Onwuegbuzie AJ, Sutton IL. A model incorporating the rationale and purpose for conducting mixed methods research in special education and beyond. Learn. Disabil. 2006; 4:67–100.

[31] Aranda R, Gonzalez-Rodenas J, Lopez-Bondia I, Aranda-Malaves R, Tudela-Desantes A, Anguera MT. “REOFUT” as an observational tool for tactical analysis on offensive performance in soccer: mixed method perspective. Front Psychol. 2019; 10:1476. 31316433 10.3389/fpsyg.2019.01476 PMC6610999

[32] Cohen J. Statistical Power Analysis for the Behavioral Sciences. New York, NY: Routledge Academic; 1988.

[33] Faude O, Koch T, Meyer T. Straight sprinting is the most frequent action in goal situations in professional football. J Sports Sci. 2012; 30(7):625–631. 22394328 10.1080/02640414.2012.665940

[34] Lago C, Casais L, Dominguez E, Sampaio J. The effects of situational variables on distance covered at various speeds in elite soccer. Eur J Sport Sci. 2010; 10(2):103–109.

[35] Hughes M, Lovell T. Transition to attack in elite soccer. J Hum Sport Exerc. 2019; 14(1):236–253.

[36] Schulze E, Julian R, Meyer T. Exploring Factors Related to Goal Scoring Opportunities in Professional Football. Sci Med Footb. 2021; 10.1080/24733938.2021.1931421. 35475738

[37] Rampinini E, Coutts AJ, Castagna C, Sassi R, Impellizzeri FM. Variation in top level soccer match performance. Int J Sports Med. 2007; 28(12):1018–24. 17497575 10.1055/s-2007-965158

[38] Gregson W, Drust B, Atkinson G, Salvo VD. Match-to-match variability of high-speed activities in premier league soccer. Int J Sports Med. 2010; 31(4):237–42. 20157871 10.1055/s-0030-1247546

[39] Zhou C, Gómez M, Lorenzo A. The evolution of physical and technical performance parameters in the Chinese Soccer Super League. Biol Sport. 2020; 37(2):139–145. 32508381 10.5114/biolsport.2020.93039 PMC7249799

[40] Vogelbein M, Nopp S, Hökelmann A. Defensive transition in soccer – are prompt possession regains a measure of success? A quantitative analysis of German Fußball-Bundesliga 2010/2011. J Sports Sci. 2014; (32):1076–1083. 10.1080/02640414.2013.879671 24506111

[41] Kubayi, A. Analysis of Goal Scoring Patterns in the 2018 FIFA World Cup. J Hum Kinet. 2020; 71(1):205–210. 32148584 10.2478/hukin-2019-0084 PMC7052713

[42] Rhodes D, Valassakis S, Bortnik L, Eaves R, Harper D, Alexander J. The Effect of High-Intensity Accelerations and Decelerations on Match Outcome of an Elite English League Two Football Team Int J Environ Res Public Health. 2021; 18(18):9913. 34574836 10.3390/ijerph18189913 PMC8471310

[43] Vázquez M, Zubillaga A, Bendala F, Owen A, Castillo-Rodríguez A. Quantification of high-speed actions across a competitive microcycle in professional soccer. J Hum Sport Exerc. 2023; 18(1): in press. 10.14198/jhse.2023.181.03

[44] Rico-González M, Rafael Oliveira R, Palucci Vieira LH et al. Players’ performance during worst-case scenarios in professional soccer matches: a systematic review. Biol Sport. 2022; 39(3):695–713. 35959320 10.5114/biolsport.2022.107022 PMC9331336

[45] Casamichana D, Castellano J, Diaz AG, Gabbett TJ, Martin-Garcia A. The most demanding passages of play in football competition: a comparison between halves. Biol Sport. 2019; 36(3):233–40. 31624417 10.5114/biolsport.2019.86005 PMC6786330

[46] Gabbett TJ. The training-injury prevention paradox: should athletes be training smarter and harder? Br J Sports Med. 2016; 50(5):273–280. 26758673 10.1136/bjsports-2015-095788 PMC4789704

[47] Malone S, Roe M, Doran DA, Gabbett TJ, Collins K. High chronic training loads and exposure to bouts of maximal velocity running reduce injury risk in elite Gaelic football. J Sci Med Sport. 2017; 20(3):250–254. 27554923 10.1016/j.jsams.2016.08.005

[48] Mohr M, Krustrup P. Comparison between two types of anaerobic speed endurance training in competitive soccer players. J Hum Kinet. 2016; 51:183–192. 28149381 10.1515/hukin-2015-0181 PMC5260561

[49] Ade JD, Drust B, Morgan OJ, Bradley PS. Physiological characteristics and acute fatigue associated with position-specific speed endurance soccer drills: production vs maintenance training. Sci Med Footb. 2021; 5(1):6–17. 35073235 10.1080/24733938.2020.1789202

[50] Oliva-Lozano J, Gómez-Carmona CD, Rojas-Valverde D, Fortes V, Pino-Ortega J. Effect of training day, match, and length of the microcycle on the worst-case scenarios in professional soccer players. Res Sports Med. 2021; 4(3):1–14. 10.1080/15438627.2021.1895786 33657955

[51] Martin-Garcia A, Castellano J, Diaz AG, Cos F, Casamichana D. Positional demands for various-sided games with goalkeepers according to the most demanding passages of match playin football. Biol Sport. 2019; 36(2):171–80. 31223195 10.5114/biolsport.2019.83507 PMC6561222

[52] Maffulli N, Kakavas G, Maliaropoulos N, Gabbett T, Mitrotasios M, Van Dyk N. A 90 Minute Soccer Match Induces Eccentric Hamstring Muscles Fatigue. Muscles Ligaments Tendons J. 2021; 11(2):318–323.

[53] Rhodes D, McNaughton L, Greig M. The temporal pattern of recovery in eccentric hamstring strength post-soccer specific fatigue. Res Sports Med. 2019; 27(3):339–350. 30296168 10.1080/15438627.2018.1523168

[54] Bradley PS, Carling C, Archer D, Roberts J, Dodds A, Di Mascio M, Paul D, Diaz AG, Peart D, Krustrup P. The effect of playing formation on high-intensity running and technical profiles in English FA Premier League soccer matches. J Sports Sci. 2011; 29(8):821–830. 21512949 10.1080/02640414.2011.561868

[55] Carling C. Influence of opposition team formation on physical and skill-related performance in a professional soccer team. Eur J Sport Sci. 2011; 11(3):155–164.

[56] Castellano J, Blanco-Villasenor A, Alvarez D. Contextual variables and time-motion analysis in soccer. Int J Sports Med. 2011; 32(6):415–421. 21590641 10.1055/s-0031-1271771

[57] Dalen T, Ingebrigtsen J, Ettema G, Hjelde GH, Wisløff U. Player Load, Acceleration, and Deceleration During Forty-Five Competitive Matches of Elite Soccer. J Strength Cond Res. 2016; 30(2):351–359. 26057190 10.1519/JSC.0000000000001063

[58] Akenhead R, Harley J, Tweddle S. Examining the External Training Load of an English Premier League Football Team With Special Reference to Acceleration. J Strength Cond Res. 2016; 30(9):2424–2432. 26817740 10.1519/JSC.0000000000001343

[59] Carling C, Bradley P, McCall A, Dupont G. Match-to-match variability in high-speed running activity in a professional soccer team. J Sports Sci. 2016; 34(24):2215–2223. 27144879 10.1080/02640414.2016.1176228

